# Investigating the Impact of Online Abortion Myths on Health Care Providers and Advocates: Interview Study

**DOI:** 10.2196/77050

**Published:** 2026-09-16

**Authors:** Rachel E Moran, Julia Simoes, Taylor Agajanian, Izzi Grasso, Anna Beers, Amanda Lock Swarr, Emma Spiro, Anna Swan

**Affiliations:** 1 Center for an Informed Public, Information School, University of Washington, 4000 15th Ave NE, Seattle, 98195, United States, 1 2132227337; 2 Department of Communication, University of Washington, Seattle, United States; 3 School of Communication, Northwestern University, Chicago, United States; 4 University of North Carolina at Chapel Hill, Chapel Hill, United States; 5 Department of Gender, Women & Sexuality Studies, University of Washington, Seattle, United States

**Keywords:** abortion, health myths, health information seeking, social media, health misinformation

## Abstract

**Background:**

Abortion is a common and safe medical intervention with a long history of practice in the United States. Despite this, inaccurate and misleading information persists, including falsehoods about the accessibility, legality, safety, and lived experience of abortion-related health care. Further, the prevalence and circulation of abortion myths online and offline have intensified following the overturning of Roe vs Wade in May 2022. While myths surrounding abortion have been widely documented, limited research has examined how these myths shape the everyday work of abortion health care providers and advocates, particularly in relation to patient–practitioner shared decision-making (SDM).

**Objective:**

This study examines how abortion myths affect abortion health care professionals—including practitioners in states where abortion access is legal, and advocates connecting patients with care in states with limited abortion health care. Through interviews with advocates and professionals, we document the nature of circulating myths, the common information sources where myths are being spread, and the routes professionals take to debunk myths when they arise, or, similarly, the barriers that limit their ability or desire to debunk abortion myths.

**Methods:**

We conducted in-depth qualitative interviews with abortion health care professionals across the WWAMI (Washington, Wyoming, Alaska, Montana, and Idaho) medical region. Interviews explored the types of abortion myths encountered in clinical settings, how providers respond to misinformation during patient interactions, and the perceived impact of myths on care delivery and SDM processes. Data were analyzed using an iterative constant comparative thematic analysis of the interview transcripts.

**Results:**

Participants reported encountering a wide range of abortion-related myths from predominantly online (social media) and some offline sources, including misinformation about safety, legality, fertility impacts, and procedural experiences. Providers described dedicating substantial time to correcting misinformation, addressing fear and confusion, and rebuilding trust within clinical encounters. Myths were found to complicate SDM by shaping patients’ expectations, emotional responses, and perceived options for care, in addition to taking up time and resources for providers of care to counter damaging myths.

**Conclusions:**

Abortion myths have tangible effects on clinical practice and SDM, placing additional communicative and emotional labor on providers and potentially undermining patient autonomy. Addressing misinformation is therefore critical not only for public understanding but also for supporting equitable, patient-centered abortion care in a post-Roe landscape.

## Introduction

### Background

Abortion is a common and safe medical intervention with a long history of practice within the United States; yet, misleading information around abortion (and pregnancy viability more broadly) persists [1,2]. Inaccurate information about abortion pertains to the realities of abortion health care (ie, information about medical interventions, medication, and aftercare) [3], falsehoods about the long-term impacts of having an abortion, and misleading information around the legality of, and access to, abortion health care [4]. The overturning of abortion protections upheld by Roe vs Wade in the 2022 Dobbs vs Jackson Women’s Health Organization (“Dobbs” for short) has exacerbated the conditions of uncertainty under which rumors and misinformation thrive [5], leading health researchers to call abortion misinformation “the next infodemic” [6]. Accordingly, given the advent of digital information-seeking for health, individuals seeking abortion information online may be met with a polluted information environment in which it is difficult to parse between falsehoods and scientific truth. Difficulties in information-seeking undermine an individual’s ability to make informed health decisions and have additional negative ramifications for their relationships with health care providers.

To better understand the ramifications of online (and offline) health myths on patient-practitioner relationships, this paper examines the impact of abortion-related myths post Dobbs from the perspective of professionals involved in abortion health care and related advocacy, including abortion practitioners and advocates. Accurate knowledge is vital for shared decision-making (SDM) between patients and practitioners to occur, with persistent myths undermining the ability of practitioners to establish relationships of trust with patients and base decisions on a mutually agreed-upon knowledge foundation. Through in-depth interviews, we explore the types of myths health care professionals encounter and how they navigate abortion-related myths when they arise, and document their impacts. Our analysis offers insights into the interconnection among legal confusion, uncertainty, and the spread of misinformation, and highlights productive routes health care professionals use to counter the harmful effects of health myths and provide a foundation for SDM.

### Extant Research

#### Health Misinformation

The spread of misleading health information is a longstanding concern for public health professionals and for science communication more broadly [7]. Further, the politicization of health-related topics has exacerbated the spread of misleading information [8], reducing the effectiveness of science communication around health and leading to significant public health impacts [9,10]. This is particularly acute in relation to abortion health care [11,12]. The overturning of historical protections of abortion access under Roe vs Wade marks a “new legal quagmire” for abortion access [13] and renews public conversation and contestation about abortion health care. Moreover, the Dobbs decision came at a time of renewed political and legislative attacks on bodily autonomy that leverage scientific misinformation as core evidence [14].

Extant research into the spread of misleading health claims primarily uses the term “misinformation” to capture the unintentional spread of nonfactual health information and “disinformation” to capture nonfactual information intentionally spread to mislead [15]. Similarly, public health scholars have used the term “myth” to describe the spread of health-related statements that are widely disseminated but have either no supportive scientific evidence or strong evidence to the contrary [16]. Given the politicization of the term “misinformation” and the nuances of scientific information that cannot necessarily be binned into the dichotomy of “true” or “false” [11], we use the term “abortion myths” throughout this work. Further, “myth” captures information that can be seen as misleading without attaching intentionality to its spread. Within the context of abortion-related information, there have historically been clear instances in which institutions have weaponized false information to intentionally discourage pregnant people from seeking out abortions. However, the complexities of the online space mean information provenance and the intentions of every poster and/or sharer of information are difficult to track. Accordingly, while we (and our interviewees) often refer to information as “misleading” given its nonfactual nature, the term “myth” affords space to avoid specific claims on intention.

#### Abortion Myths

The scope of abortion-related myths and their spread in online and offline environments has been well-documented. Recent research, for example, highlights the prevalence of misleading information about abortion and herbal “at-home abortion remedies” on video-sharing site TikTok (ByteDance) [17]. Earlier work explored the spread of inaccurate information related to abortion and breast cancer, infertility, and mental health problems by pregnancy crisis centers [18,19]. Further research has highlighted the role of abortion myths in pro- and antiabortion advocacy. The work by Gantt-Shafer [20] examines the impact of myths on the overall climate around abortion, finding that media representations or misrepresentations of abortion can normalize rage as an acceptable response to the existence of abortion and further the demonization of people who seek out this form of health care.

Documenting the perspectives of health care providers is particularly necessary post Dobbs as abortion health care professionals navigate a new legal climate around abortion health care [21]. Myths that arise in conjunction with new laws exacerbate confusion around legality. Location-specific research points to these complexities. For instance, a report from the Idaho Coalition for Safe Healthcare found that Idaho—a state that outlawed abortion following the Dobbs decision—lost 22% of its obstetrics and gynecology (OB-GYNs) in the year following the decision. Doctors in the state have argued that the scope of Idaho’s abortion ban is unclear, creating confusion on “what care they can and can’t provide patients” [22]. As such, it is important to capture how legal confusions and the rumors that arise out of this confusion impact provider well-being.

Accordingly, in the wake of the Dobbs decision, a wave of perspectives papers in public health and medical journals have called for the active participation of health care professionals in reproductive justice advocacy work [23,24]. Within this has been a call for practitioners to consider illuminating abortion myths as part of the professional obligations of health care providers [25]. However, little research exists documenting the specific impacts of abortion myths these health care providers encounter and how they attempt to debunk misleading information about abortion when it arises.

#### Theories of SDM

While SDM has become the norm for patient-practitioner relationships within the United States [26], there is no singular definition describing the process. According to Resnicow et al [27], most models of SDM consider it a “collaborative process by which both the patient and health care provider contribute to medical decisions, with patients being full partners in decisions about their care...” In defining the underpinning theoretical mechanisms supporting SDM as a relational care route, Resnicow et al, present two relevant models—self-determination theory (SDT) and the difficulty × motivation matrix. SDT [28] proposes a continuum of motivational regulation—ranging from controlled motivation, that is, behavioral change emanating from external pressure and guidance, to autonomous motivation—self-determined change through internal motivation [28]. Further, SDT advances three fundamental human needs—competence, relatedness, and autonomy—that, once met, enhance behavior change by affording more autonomous motivation. Building on this, Resnicow et al [27] point toward their previously established difficulty × motivation matrix [29] to explain how the effectiveness and style of SDM needed may also be impacted by the degree of difficulty the behavioral change requires. More complex, repeated, and long-term health behaviors, for example, smoking cessation, dietary change, require more patient autonomy than one-off or less complex health decisions. Taken together, the theoretical underpinnings of SDM highlight the utility and necessity of patient autonomy in health decisions and foreground the role of information access (foundational for patient competency) in effective SDM.

Empirical studies of abortion health care examine the potential role of SDM in ensuring effective health outcomes and patient satisfaction [30]. Fagot [30] argues that SDM is an effective care strategy for patients seeking medication abortion, but one that is “multifaceted and difficult to implement in health care settings.” Difficulty arises due to systemic barriers to accessing abortion care, such as cost, logistical barriers, and information overwhelm. Studies outside the US context confirm similar issues. Research by Frederico et al [31] in Mozambique highlighted how gender and power inequalities and a lack of knowledge at the national level surrounding the legality of abortion limit women’s autonomy in decision-making around abortion health care. Shared-decision making thus requires health practitioners (and broader advocates) to uncover and attend to barriers that undermine the ability of patients to autonomously engage with health information and thus effectively uphold their role within the shared patient-practitioner decision-making relationship. One such barrier is increasing distrust in medical professionals [32], which is further connected with the rise of medical misinformation as individuals look elsewhere (increasingly online) for answers to their health queries, and the persistence (or perception) of health misinformation causes further distrust in health care providers [12]. However, there exists a dearth of research examining (1) the spread of abortion-related myths in the US-context post Dobbs, and (2) exploring how such myths impact practitioners in their ability to foreground SDM in their patient relationships.

To address current gaps in research, this project seeks to answer the following research questions (RQs):

- RQ1: what types of myths do providers and advocates encounter?
- RQ2: how do they navigate myths when they arise?
- RQ3: what impacts do they see myths having on abortion health care?

## Methods

### Sampling and Recruitment Methods

The research team conducted 14 semistructured, in-depth interviews with abortion health care providers and advocates between October 2023 and January 2024. Interviewees were recruited via email through snowball sampling [33] using email listservs and personal connections with researchers and practitioners in the WWAMI (Washington, Wyoming, Alaska, Montana, and Idaho) medical region, plus the neighboring state of Oregon. The inclusion criteria for interviews defined appropriate interviewees as individuals working or who have worked professionally within the US abortion space—either as licensed medical or adjacent health care professionals or reproductive rights advocates affiliated with professional organizations—who attest to having been impacted in some way by the spread of false or misleading information about abortion. Meeting the latter criteria was determined within the recruitment process through initial email advertisements (asking for participants who had encountered misinformation about abortion within their professional work) and in confirmation through sharing (and confirmation of awareness) of the research objectives within informed consent. We faced difficulties in recruiting health care providers in states, notably Idaho, where abortion access is banned. To expand our potential interview sample, we recruited advocates working within abortion and health care organizations to advance abortion access, in addition to health care practitioners who either practiced abortion lawfully under their home state’s laws or provided general health care (usually OB-GYN and family-planning-related care) in banned states. Snowball sampling began with abortion health care providers within our university-affiliated hospital system, who then referred us to email listservs for abortion-related professionals and directly connected us with practitioners from other states within their professional networks. The email listservs used were aimed at either members of professional organizations associated with abortion and related health care or broader organizations and collectives engaged in work to advance abortion rights. Our resultant sample (Table 1) includes health care practitioners—in the main OB-GYNs and 2 primary care physicians and 2 abortion advocates—both working in abortion-specific organizations connecting individuals in banned states to health care and engaging in legislative advocacy.

**Table 1.** List of participants with state location and professional role.

Participant ID	State	Professional role
001	MT	Health care practitioner
002	WA	Health care practitioner
003	WA	Health care practitioner
004	WA	Health care practitioner
005	OR	Health care practitioner
006	MT	Health care practitioner
007	MT	Health care practitioner
008	WA	Health care practitioner
009	WY	Abortion advocate
010	MT	Health care practitioner
011	ID	Health care practitioner
012	WY	Health care practitioner
013	ID	Abortion advocate
014	AK	Health care practitioner

### Ethical Considerations

This project was reviewed by the Institutional Review Board at the University of Washington and deemed exempt from federal human participants review (STUDY00018372). The University of Washington’s Human Subjects Division determined that this interview study qualified for exempt status (category 2). Accordingly, formal written informed consent was not obtained. Instead, interviewees were provided with a statement blurb about the purpose of the research project, how data (interview transcripts) would be obtained, anonymized, and analyzed, and contact details for this study’s team (Multimedia Appendix 1). Study details were emailed to potential participants before scheduling an interview time, and we repeated this at the beginning of interviews (before recording), at which time this study’s team repeated that participants could remove themselves and their data from this study at any time (Multimedia Appendix 1). Given the sensitive nature of this study’s subject matter, interviewee privacy was of the utmost importance. Interviewees were given a number identifier that was not attached to their name and/or contact information, and only two biographical details—their state and a generalized professional title (Table 1)—were collected to ensure confidentiality. All participants were compensated for their time with a US $50 gift card.

### Research Team Positionality

All interviews were conducted by the lead author, with a secondary interviewer from the broader research team. The research team consists of social scientists from different disciplines researching issues related to health information-seeking, reproductive health and advocacy, and social media. Researchers were all PhDs trained in qualitative research methods, including interviews and thematic analysis. All of the research team were employed by the University of Washington either as full-time academic researchers or as doctoral students and research assistants. Researchers within the team identify as either female or nonbinary.

### Interview Protocol

Interviews were semistructured, following a general agenda with intentional space for participants to direct conversation based on what they wanted to share. A protocol was developed by the research team that focused on capturing the professional experiences of interviewees in relation to the informational climate surrounding abortion to answer the core RQs related to common myths (RQ1), how they navigate these myths in their professional work (RQ2), and the broader impacts they see abortion myths as having on abortion health care (RQ3). Specific questions in the agenda pertained to experiences of abortion myths within patient interactions, considerations of public knowledge around abortion health care and prominent myths and misconceptions, experiences of patient information-seeking about abortion, and strategies interviewees deploy when they encounter abortion myths in their patient interactions (see Multimedia Appendix 2 for interview protocol). Interviews were conducted via Zoom (Zoom Communications, Inc) and lasted, on average, 45 minutes.

Audio recordings were taken for transcripts to be obtained and then destroyed. The research team met to debrief after each interview and discuss emergent themes before formal coding. We continued interviews until the research team agreed that we had achieved data saturation [34], that is, we reached a point of “informational redundancy” [35], where new interviewees provided similar answers to several prior interviews.

### Data Analysis

Researchers conducted a grounded thematic analysis of the interview transcripts [36], starting from the interview dataset to ascertain common themes, then codifying themes into a specific codebook applied to interview transcripts to gauge prevalence, capture any contradictions or nuances between perspectives, and surface appropriate descriptive quotes from interviews. An initial team of 4 researchers used qualitative data analysis software Atlas.TI to open-code the full set of transcripts to identify initial themes and points of interest. In the open-coding phase, researchers read through transcripts and labeled data (usually single sentences or brief paragraphs) of interest to the core RQs. Labels consisted of in vivo or descriptive codes. The full research team then met and used visual collaboration software Miro to undertake a collaborative clustering activity in which researchers colocated similar codes, identified salient themes from the clusters using a constant comparative method, and discussed connections between clusters. A smaller group of researchers then used the results of the clustering activity to form the basis of a codebook (Multimedia Appendix 3), which was used by the full research team to code all 14 transcripts. The findings section highlights the most pertinent themes that emerged from this analysis.

## Results

### RQ1: What Types of Myths Do Providers and Advocates Encounter?

Throughout the interview process, providers and advocates highlighted several central myths they encounter in their work that are predominantly procedural (around reproductive health and abortion as a medical intervention) or legal. Table 2 highlights the core thematic codes emergent from interview analysis regarding the types, sources, and routes to combating the central myths encountered by providers and advocates.

**Table 2.** Core themes emergent from interview analysis regarding major myths, sources of myths, and routes to combating myths.

Core theme	Subthemes	Description
Sources of misinformation	Offline	Descriptions of offline sources of misinformation, for example, friends and family, church, legal framework, etc
Sources of misinformation	Online	Description of online sources of misinformation (not social media), for example, Google, websites
Sources of misinformation	Social media	Description of social media sources of information, for example, TikTok, Facebook (Meta), X (formerly known as Twitter), etc
Sources of misinformation	Crisis pregnancy centers	Explicit mentions of the role of crisis pregnancy centers in “care” giving and information-seeking
Types of misinformation	Procedural misinformation	Any misinformation related to abortion procedures and the risks and pain involved
Types of myths	Future risks and impacts	Misinformation related to potential future impacts of having an abortion, for example, breast cancer, mental health, and infertility
Types of myths	Legal misinformation	Misinformation related to the legality of getting, delivering, or assisting with an abortion
Types of myths	Misinformation in options counseling	Misinformation given during counseling, for example, around options available to patients
Types of myths	Misinformation regarding the reputation of the reproductive health field	Mentions of misinformation related to the reputation of health care providers (providers are “bad people”) and institutions such as Planned Parenthood
Strategies for combating myths	Barriers to addressing misconceptions	Mentions of the barriers that exist around addressing abortion misconceptions (ie, limited time with patients)
Strategies for combating myths	Communication strategies	Mentions of the ways providers communicate with patients about abortion, their misconceptions about abortion, and how they build interpersonal trust with patients
Strategies for combating myths	Evidence-based information sharing	Mentions of how providers share information with patients as a way to combat misconceptions
Strategies for combating myths	Using social media	Mentions of the use or potential of social media to combat misconceptions among a broader audience
Strategies for combating myths	Seeking out different perspectives	Intentionally seeking out differing perspectives about abortion to build understanding about what distinct ideas or misconceptions exist around abortion

Procedural myths and misconceptions emerge mainly around the safety of abortion, both medication and procedural. Providers stressed that while much of the discourse around abortion presents it as a painful, dangerous, or risky procedure, it is exceedingly safe, especially compared to pregnancy, which can be very high-risk. One provider noted:

*I think also there’s a lot of disinformation about the safety of abortion in general. Where we know that it is much, much safer than continuing a pregnancy, and there’s generally no significant risk of infertility or infection, or significant bleeding that is associated with having an abortion.*

[D004]

Additionally, multiple providers noted that there are large gaps in public knowledge about medication abortion—mifepristone and misoprostol—which can be safely self-administered. D008 argued:

*I think there’s a lot of belief about abortion needing to be medicalized. And I think, like there are like organizations and then also people in the family planning community providing this care, who are trying to do more work and supporting people who choose to have abortions on their own as far as self-sourcing medication abortion, or self-managing. But I think there’s—I guess I worry about patients who choose that even coming into the system with complications, or, you know, bleeding, or whatever it might be and then providers being like, Oh, you’re so irresponsible for doing that. Whereas, like actually, abortion is really safe and you know, there are studies showing people do know how far along they are in pregnancy. They can take a questionnaire to self-assess. You know their risk for ectopic and like, safely have self-managed abortion.*

While procedural myths and misconceptions most commonly come from patients, myths around legality are also pervasive among the practitioners and advocates themselves—including myths about what was or was not legal and where. D003 noted:

*Yeah, I mean, I think that it makes, it may impact, not just patients, but colleagues, staff. You know, misunderstandings about the legality of abortion like, persist in my own department about like what Washington State’s laws mean, in fact. And so like, if we can’t understand it as physicians and clinicians like, how are we gonna expect, you know, like other folks to understand it, if you know, if it’s like, literally part of our job.*

However, while mounting legislation in some banned and restricted rural states often leads to confusion and/or misinformed assumptions about access, D009 also noted some of these assumptions predated the Dobbs decision and were rooted in preexisting rural community attitudes about abortion:

*It’s understandable that people don’t quite know if it’s legal or not. But even before that a lot of people just had an assumption. Oh, I didn’t think it was legal in Wyoming, you know.*

Interviewees were also asked about the source of the myths and misconceptions they encountered in their industry and in their encounters with patients. Offline sources were identified as common sources of myths around abortion by our participants, with crisis pregnancy centers (CPCs) noted most frequently, followed by interpersonal relationships and other offline sources. On CPCs, 1 provider noted:

*I mean, it’s extremely angering whenever I even talk about them, because I do agree like not being a legal person myself, I don’t completely understand the legal reasons they’re allowed to exist and provide this false counseling and information to patients regarding like patients own health. Like people, are literally like pretending to put an ultrasound on, don’t even know how to operate an ultrasound machine like lying to patients about how far along they are in pregnancy like trying to convert them to religions.*

Online sources were also highlighted by interviewees, including media such as informational websites (sometimes run by CPCs), news media, and social media. Many of the providers interviewed discussed actively avoiding social media (and even online news media in some cases) for several reasons, including a lack of free time but predominantly due to the volatility of social media and an impression that the amount of online misinformation would be overwhelming. One provider noted:

*I haven’t been on social media, for you know, many, many years, and so, in fact, that probably means that I’m not exposed to, you know, sources of kind of misinformation that other people are, and certainly patients might be exposed to.*

[D006]

The aversion to online media expressed by participants could help explain why offline sources emerged as the leading source of abortion myths across interviews, despite extant research documenting the importance of social media in information-seeking. Moreover, it highlights a potential gap in knowledge for practitioners, as protecting their own mental well-being through staying away from social media also results in their (known) lack of awareness of the full extent of myths circulating on online platforms. Despite their personal avoidance of social media, interviewees did highlight the emergence of social media as a source of reproductive health myths from interactions with their patients, with specific mention of TikTok and Reddit.

In talking through the types of myths that were pervasive in their practice and/or geographic communities, interviewees reasoned why certain myths retained salience within the community or within the US more broadly, such as religion and language. The importance of religion in the framing of life and the sanctity of life led to a belief in myths around gestational limits, the availability of certain procedures, future fertility, and health, in addition to stigma and shame. One provider argued:

*I think fear tactics are the most impactful where there is so much misinformation about the safety of abortion, and especially in religious worlds, the idea that abortion is a sin, or that someone will go to hell if they have an abortion, I think that is extremely impactful in people of faith, and in making them either feel extremely confused or, or making the decision very difficult for them, or actually making them change their decision. And I think that that is very unfortunate, and very negatively impactful both for the patient as well as for the providers taking care of them.*

[D004]

Interviewees also discussed the weaponization of language around abortion, especially the use of sensationalized nontechnical terms which they saw as contributing to misleading narratives of abortion health care. For example, D014 discussed the use of the term “partial-birth abortion,” which they implored is “not even a thing.” According to The Guttmacher Institute, the term “partial-birth abortion” is a medically inaccurate term coined by the National Right to Life Committee [37]. The Cornell Legal Information Institute defines the practice as “an abortion in which the person performing the abortion— (A) deliberately and intentionally vaginally delivers a living fetus...for the purpose of performing an overt act that the person knows will kill the partially delivered living fetus; and (B) performs the overt act, other than completion of delivery, that kills the partially delivered living fetus” [38].

### RQ2: How Do They Navigate Myths When They Arise?

Interviewees expressed hesitancy at debunking abortion-related myths through outright fact-checking. D005 argued that they “don’t like to try to fact check them [patients]. I just try to kind of nudge them towards openness.”

Part of this hesitancy can be attributed to the intersection of the salience of abortion myths with growing distrust in medical institutions. Several interviewees highlighted the parallels between COVID-19 and reproductive health myths in the postpandemic climate, emphasizing an antigovernment, antiregulatory stance that contributes to myths about public health—propagated through nonmedical, sometimes adversarial sources. D005 discussed changing their entire approach when talking to patients under this newfound climate of medical distrust, while D008 noted their difficulty gaining back trust despite being an expert in their field:

*And so there’s the unknown. And then individuals hear things, and they think that’s the truth. And then it’s just misinformation. And then I think you also have a community of people that might be anti-medicine, anti-physician ever since Covid. And if anything does come out that has some type of credibility behind it, it is immediately dismissed because they just can’t even go there because of lack of trust. And that’s a whole other thing is trying to gain back trust of people to hear us to say, this is, this is how it works, this is how it is.*

Moreover, D008 argued institutional distrust may be particularly salient for marginalized patients seeking care, noting:

*I think like fear is a much stronger emotion than others. And so I think it’s easy if you hear something that instills fear in you like I think it’s easy to like get stuck on that idea or that thought and focus on that especially if you are somebody who might not even trust the healthcare system like, who can you trust, you know, like, I think, patients for good reasons, like because of historical coercion, you know, forced sterilization, other things the healthcare system has perpetuated on marginalized populations.*

Such findings echo long-standing concerns around medical mistrust and its impact on maternal medicine, particularly within Black communities [39], in addition to rising concerns about how the overturn of Roe vs Wade will impact patient-provider trust [40].

Instead of directly debunking myths, interviewees advocated that a core part of their role (in health care provision and advocacy) was to understand the motivations underpinning the sharing of and belief in myths and to redirect patients toward better information. Interviewees saw this approach as a route to “non-judgmental care” (D006). To understand patients’ information-seeking and reorient them away from misleading information, interviewees engage in several communications strategies, including providing evidence-based information from a variety of vetted online and offline sources and engaging in motivational interviewing (MI) techniques. Several interviewees highlighted the utility of providing patients with handouts or directing them to online resources in countering myths and allowing patients to access accurate information in their own time. Interviewees named the ACOG (American College of Obstetricians and Gynecologists), the National Abortion Federation, Planned Parenthood, and a host of locally specific abortion organizations as useful sites they directed patients toward. Several interviewees highlighted how overwhelming information-seeking and decision-making around abortion health care can be and how this necessitated that practitioners provide additional resources beyond the clinical appointment. As one interviewee argued:

*I usually honestly print out an ACOG bulletin for them [patients]. I feel like that’s helpful for me to be like, “hey this information is overwhelming when you’re going through a conversation like this on a clinic visit”...I’ll give them information from ACOG and then usually try to have them come back in a few days.*

[D007]

In addition, MI, that is, asking open questions to explore a patient’s perspectives and underlying rationale, emerged as a core practice across interviews. Interviewees summarized MI as follows:

*I’ll ask a little more about that belief, how they came to that belief, what their source was, how they feel about that particular piece of information.*

[D004]

MI laid a foundation upon which practitioners felt more comfortable to dispel abortion-related myths. Interviewees saw the practice as less dismissive than simply fact-checking and afforded them the ability to build trust with their patients. Moreover, MI allowed abortion health care professionals to identify which patients are likely to change their minds, and when. D001 argued:

*I basically do motivational interviewing like: “how willing and ready are you to think differently on this? Zero? Okay, moving on, not going to waste my breath. It’s not worth my energy...[I have] limited time and resources with a patient that you have to choose to spend them wisely.”*

However, interviewees also named several complicating factors when navigating abortion-related myths when they arise in patient interactions. As highlighted previously, limited time with patients curtailed their ability to debunk myths both in terms of logistics—short consultation periods meant that debunking myths is not always a priority in caregiving—and in limiting the ability of health care professionals to build the trust necessary for debunking to be impactful. D003 summarized the impact of this limited interaction, explaining why it was unsurprising that patients trusted health care professionals less than those in their everyday circle:

*I might be somebody with some fancy letters behind my name, but they’re just meeting me and so if their auntie, their religious leader, their sibling, their best friend, you know some super trusted person. Like that carries a ton of weight with people and so it might just be that they may or may not believe me.*

Further, health care practitioners spoke about not always being aware of what information patients encountered, mostly due to them intentionally staying away from social media. The (nonmedical) abortion advocates interviewed were more aware of information spreading on social media, whereas common across the (medical) practitioners interviewed was a desire to stay away from social media and avoid abortion-related news coverage due to the mental health toll of being immersed in the subject matter in both their professional and personal lives. Practitioners knew from patient interactions that social media is playing a large part in information-seeking but had limited direct knowledge of the kinds of information that exist online. In these conversations, several interviewees named TikTok as a source of patient information-seeking. D014 argued:

*TikTok is now the new doctor, [it] used to be Dr. Google. Now it’s Dr. TikTok. I don’t know how to dance! How am I supposed to compete with TikTok now that’s where people are getting their information?*

As such, interviews demonstrated uncertainty among practitioners in engaging directly in fact-checking of abortion-related myths, predominantly because they do not always know the extent of misleading information a patient may have been exposed to and have not had the chance to sufficiently build trust and rapport with patients for fact-checking to be effective. D008 encapsulated such difficulties:

*I think as a provider...I only know the surface level of what patients are even wondering about, because I wonder if patients even feel comfortable sometimes asking the questions they’re really thinking.*

### RQ3: What Impacts Do They See Myths Having on Abortion Health Care?

Several interviewees noted how their perspectives were limited to the patients they encountered and that a hidden population of pregnant people who choose not to seek out health care providers or abortion advocates may be impacted by misleading information. D004 characterized antiabortion messaging as misinformation intentionally targeted to dissuade people from having abortions:

*I think that there’s a very intentional conscious output of misinformation that’s driven by people who are against abortion access and do not want people to be having abortions.*

Further, interviewees highlighted how, despite sharing misinformed views on the safety of abortion, the potential of lasting consequences, the legality, and even the pain associated with procedures, patients still chose to go forward with terminations.

*But patients have those fears because of that disinformation and present for care anyway, because they do not want to be, and cannot be, pregnant at that time.*

[D003]

For those who do access care, abortion myths may act to amplify fear, cement stigma, and increase the emotional impacts of abortion health care [41]. In addition to the impacts of negative sentiments associated with abortion on patients, interviews highlighted how abortion myths have similarly led to negative implications for providers. Providers are subject to attacks on their credibility, on a professional and individual level, that emerge from misunderstandings about the nature of their abortion-related work. In addition to negatively impacting their well-being, such misinformed attacks have also led health care professionals to avoid abortion-related work. D001 argued that they would love to do abortions, but it “would be professional suicide where I’m at.”

Interviewees spoke extensively about their physical safety and ensuring the physical safety of fellow abortion health care providers and advocates. One interviewee described a recent incident in which someone used a “shotgun to blast two holes in our [the clinic’s] front door” (D006), another provider’s clinic was burned down by an arsonist just before it was due to open (D009), and yet another recounted how their apartment had been broken into, trashed, and abortion-related messages were left on a whiteboard (D010). Interviewees saw such acts of violence and intimidation as associated with the sustained spread of myths within the antiabortion movement. Long-cemented myths about the nature of abortion procedures and about fetal development and viability have radicalizing effects for some, driving more extreme opponents to target abortion health care providers with violence and intimidation. Resultantly, across interviews, professionals highlighted the mental and physical toll of working within abortion health care and fears for their physical safety. In addition to concerns about physical safety, interviewees feared that the potential for violence could drive health care professionals out of abortion work. D006 argued:

*That’s [violence] the kind of thing that really has a potential for causing disruption. Right? If my colleagues quit because they’re fearful for their own safety, then we can’t see patients.*

At the extreme, abortion myths contribute to the escalation of antiabortion violence, and in the everyday, they reinforce long-standing stigma around abortion, abortion-seekers, and abortion providers [42]. D001 described the community shaming they and their colleagues experienced, despite not performing abortions, because of misled beliefs about abortion health care:

*I literally have patients who won’t see me because they think I actually do abortions every day. My mentor here had someone stand up in church and yell at her and start screaming that she did abortions because she did D&Cs for miscarriages.*

Myths about abortion health care encompass a wide array of reproductive health topics and, notably, result in stigma around lifesaving, often emergency, care necessary for miscarriages. D001 described an interaction where they explained care options to a patient experiencing a stillbirth at 31 weeks, resulting in the patient sobbing that they did not “want to have an abortion” because they were equating the dilation and curettage procedure needed to safely complete a miscarriage with an elective abortion.

In contemplating the impacts of abortion myths and their interaction with societal stigma, interviewees also highlighted how these myths act as barriers to care because of the time needed to debunk myths and settle associated feelings of stigmatization.

*If you spend more time sort of working to dispel this [stigma], this might delay someone getting an abortion*.

[D002]

Delaying an abortion is not necessarily negative if patients are early enough in their pregnancy and live in a state that affords them options. However, recent and ongoing legal changes around gestational time limits complicate options for pregnant people; as D007 summarized, “if it turns into a second trimester, then they [may not] be able to find a place that does that.”

The impact of abortion myths thus requires consideration of the legal framework for abortion, particularly post Dobbs. In addition to novel legal myths around abortion arising in the complicated landscape following the overturning of Roe vs Wade, interviews highlighted an uptick in abortion-related conversation, resulting in the potential for further amplification of abortion myths. D004 posited:

*I don’t know if ... there’s necessarily more accurate information out there than there used to be, but there’s definitely just more information in general, [more] talking about abortion.*

## Discussion

### Principal Findings

Interviews captured abortion professionals’ perspectives on the types of myths circulating about abortion health care and the impact they are having on their work. The post-Dobbs context has brought to the fore a novel arena for abortion-related myths focused on legality. Interviewees expressed concern over the rapidly altering legal landscape around their work and how this leaves both the public and abortion professionals unclear on the legalities of specific abortion-related (and broader) procedures. Given this dynamic uncertainty, an undeniable information void has emerged for those seeking up-to-date information about the legality of abortion in their specific location.

An exploration of the nature of abortion myths in information-seeking, attempts to mitigate misleading information in the clinic, and the perceived impacts of abortion myths holds significant insights for models of SDM. Previous examinations of SDM in clinical settings explore the patient-side of SDM dynamics, in addition to the role of the practitioner in allowing for patient autonomy [43,44]. Interview findings cement the difficulties of engaging in SDM, and in particular, highlight how misleading and false information can undermine the foundations of patient competence, relatedness, and autonomy [29] central to SDT and thus at the core of effective SDM. In an information environment flooded with abortion-related myths, the ability of patients to engage autonomously in care-related discussions is further eroded, and the role of practitioners in debunking myths may complicate a patient’s sense of autonomy. Moreover, uncertainty around abortion myths (specifically about legality) among practitioners highlights a need to attend further to the practitioner-side of SDM dynamics and not take for granted the competence and sense of autonomy felt by practitioners in their navigation of patient care plans.

Conversations also looked to understand practitioners’ and advocates’ perspectives on the information-seeking environment for abortion health care. Two key takeaways emerge from the analysis findings. First, online sources—including new social media sites such as TikTok—are primary sites for abortion-related information. However, professionals express concern over the credibility of information patients are accessing on these platforms, given the prevalence and diversity of myths they encounter in their work. Coupled with the fact that many interviewees acknowledged that they do not use social media, often purposefully to avoid personal overexposure to stigma and the negative emotional toll of encountering abortion myths, means they can only be reactive within patient interactions as they are not personally aware of the social media landscape. Emergent academic research is instructive in learning about the kinds of myths—for example, around safety, how to access and use oral abortive medications, legality, pain, etc [2,6]—but more work needs to be done to provide abortion health care professionals with knowledge about viral myths that does not require them to take on the individual burden of being active on social media. Further, as social media becomes a central authority for health information for patients, attending to the nature of health information accessible (and prominent) on social media platforms is crucial to building effective SDM.

Second, abortion health care professionals have developed useful communication strategies that enable productive conversations around myths. Instead of looking to directly fact-check or debunk myths presented to them, most interviewees described a communication process akin to MI—that is, asking open questions about the nature of the information presented and its utility or value to the patient. MI allows professionals to build a rapport with patients in addition to getting to the heart of why a specific abortion myth is particularly salient to that person. Accordingly, MI appears to be a core tool in enabling SDM to occur within a polluted information environment. Future research should explore how MI techniques can be strengthened through proactive knowledge of what types of myths are spreading in the digital information environment and how MI impacts a patient’s sense of autonomy in SDM relationships.

Finally, interviews capture a pernicious cycle of distrust. Distrust of abortion providers—often underpinned by stigma and/or misled views about abortion and providers—may lead information-seekers to look to online and alternative, mostly online, sources of information about abortion health care. In doing so, information-seekers encounter further myths about abortion procedures, the consequences or impacts of abortion, and the legality of abortion. These myths further fracture trust in abortion health care providers, and in the extreme, they can lead to the harassment of practitioners, as highlighted by several interviewees. Moreover, distrust at any level undermines the ability for SDM to occur, as patients are unwilling to engage in decision-making models that afford practitioners agency, and vice versa for practitioners concerned that patients cannot give informed consent without accessible, factual information. Effectively addressing distrust most often requires individual interactions [45], yet this is impossible to achieve if patients are too distrustful to talk to health care professionals about abortion, or even access health care at all.

### Limitations and Future Work

This research is necessarily limited by its focus on health care providers and advocates. Interviews capture the perspectives of abortion health care professionals, meaning that discussions of the impact of abortion myths are filtered through the experiences of these professionals, rather than directly capturing the lived experiences of those seeking abortion health care. Further, we recruited professionals with a preexisting awareness of the issue of misinformation in their field. While we anticipate that this is a common awareness within abortion health care, there may be practitioners who do not feel as acutely the impact of abortion myths on their work. As such, future research should supplement this understanding through interviews and/or survey work designed to document the spread and impact of abortion myths within patient and broader populations. In addition, interview research and subsequent analysis are grounded within the perspectives of the research team conducting this project. We recognize that our positionality, both as academic researchers and associated with our individual identities, may impact how interviewees responded to answers within the interview context and similarly biased our thematic analysis. Our research methodology is designed to reduce the impact of researcher bias, as is transparency in our reporting; however, research from outside of academia may identify additional or conflicting perspectives on abortion myths that we did not capture.

Further, as highlighted by practitioner concerns, this work only captures the perspectives of patients who access health care, excluding a large population of information-seekers who do not access abortion-related health care. Accessing this missing population is difficult, but future work should look to address this gap to gain further understanding of the impact of misleading abortion information. Finally, this work took place shortly after the Dobbs decision, wherein 13 states activated trigger laws that banned or heavily restricted abortion [46]. This immediate period can be characterized by heightened confusion, as states figure out how to enact said bans and as challenges have emerged around specific cases and restrictions more broadly. Longitudinal research should look to examine how abortion myths shift (in prevalence and nature), as the aftermath of the Dobbs decision becomes more embedded in the legal frameworks of low and no-access states.

### Conclusions

Misinformation, myths, and rumors thrive in conditions of uncertainty [47], and the legal complexity and uncertainty post Dobbs (Cohen et al, unpublished data, 2022) have created an environment ripe for the spread of novel and long-standing abortion myths. Moreover, the costs of myths within abortion health care are substantial. Interviews capture the strain abortion myths create in the ability of practitioners to pursue effective SDM with patients, including undermining trust in patient-practitioner relationships, delaying desired and/or essential health care to negative outcomes, and even causing violence and harassment directed at health care providers. The future of informed consent and effective SDM for abortion health care relies upon access to authoritative information. The current online information environment concerning abortion is muddled, with added complexity because of the idiosyncrasies of access according to geography and legality. Abortion myths are long-standing, underpinned by active disinformation spread by CPCs about the nature and impact of abortion procedures and by antiabortion activists. The resultant information environment is highly vulnerable to the spread of abortion myths that undermine patient autonomy. Accordingly, abortion health care exists as an important case study for understanding models of SDM in an era of information disorder. This project highlights how health myths undermine the conditions necessary for autonomy and self-determination within health decision-making, suggesting a need to update theories underpinning SDM to account for the realities of patient information seeking.

## Supplementary material

10.2196/77050 Multimedia Appendix 1 Consent (short form).

10.2196/77050 Multimedia Appendix 2 Interview protocol.

10.2196/77050 Multimedia Appendix 3 Codebook.

## Abbreviations

ACOG: American College of Obstetricians and Gynecologists
CPC: crisis pregnancy center
MI: motivational interviewing
OB-GYN: obstetrics and gynecology
RQ: research question
SDM: shared decision-making
SDT: self-determination theory
WWAMI: Washington, Wyoming, Alaska, Montana, and Idaho

## References

[1] Facts are important: abortion is healthcare. American College of Obstetricians & Gynecologists.

[2] Patev AJ, Hood KB (2021). Towards a better understanding of abortion misinformation in the USA: a review of the literature. Cult Health Sex.

[3] Heymann O, Odum T, Norris AH, Bessett D (2022). Selecting an abortion clinic: the role of social myths and risk perception in seeking abortion care. J Health Soc Behav.

[4] Pleasants E (2021). Challenging abortion disinformation online. UWIRE Text.

[5] Spiro E, Starbird K (2023). Rumors have rules. Issues Sci Technol.

[6] Pagoto SL, Palmer L, Horwitz-Willis N (2023). The next infodemic: abortion misinformation. J Med Internet Res.

[7] Suarez-Lledo V, Alvarez-Galvez J (2021). Prevalence of health misinformation on social media: systematic review. J Med Internet Res.

[8] Druckman JN (2022). Threats to science: politicization, misinformation, and inequalities. Ann Am Acad Pol Soc Sci.

[9] Beers A, Nguyễn S, Starbird K, West JD, Spiro ES (2023). Selective and deceptive citation in the construction of dueling consensuses. Sci Adv.

[10] Goldstein CM, Murray EJ, Beard J, Schnoes AM, Wang ML (2020). Science communication in the age of misinformation. Ann Behav Med.

[11] Godoy M (2022). Doctors and advocates tackle a spike of abortion misinformation – in Spanish. NPR.

[12] Stimpson JP, Park S, Adhikari EH, Nelson DB, Ortega AN (2025). Perceived health misinformation on social media and public trust in health care. Med Care.

[13] Kulczycki A (2022). Dobbs: navigating the new quagmire and its impacts on abortion and reproductive health care. Health Educ Behav.

[14] Lockmiller C (2023). Decoding the misinformation-legislation pipeline: an analysis of Florida Medicaid and the current state of transgender healthcare. J Med Libr Assoc.

[15] Krishna A, Thompson TL (2021). Misinformation about health: a review of health communication and misinformation scholarship. Am Behav Sci.

[16] Kessler SH, Bachmann E (2022). Debunking health myths on the internet: the persuasive effect of (visual) online communication. J Public Health (Berl).

[17] Sharevski F, Loop JV, Jachim P, Devine A, Pieroni E (2023). Abortion misinformation on TikTok: rampant content, lax moderation, and vivid user experiences. arXiv.

[18] Bryant AG, Narasimhan S, Bryant-Comstock K, Levi EE (2014). Crisis pregnancy center websites: information, misinformation and disinformation. Contraception.

[19] Bryant AG, Levi EE (2012). Abortion misinformation from crisis pregnancy centers in North Carolina. Contraception.

[20] Gantt-Shafer J (2020). “They just went after us:” reproductive justice advocacy at an abortion fund. Front Commun.

[21] Sabbath EL, McKetchnie SM, Arora KS, Buchbinder M (2024). US obstetrician-gynecologists’ perceived impacts of post-Dobbs v Jackson state abortion bans. JAMA Netw Open.

[22] Luchetta J (2024). Report shows dramatic exodus of Idaho obgyns since repeal of Roe v. Wade. Oregon Public Broadcasting.

[23] Kozhimannil KB, Hassan A, Hardeman RR (2022). Abortion access as a racial justice issue. N Engl J Med.

[24] Wilkinson B, Onwuzurike C, Bartz D (2022). Restrictive state abortion bans - a reproductive injustice. N Engl J Med.

[25] Carroll S, Joshi D, Espey E (2022). Physicians and healthcare professionals as advocates for abortion care and reproductive choice. Curr Opin Obstet Gynecol.

[26] Légaré F, Thompson-Leduc P (2014). Twelve myths about shared decision making. Patient Educ Couns.

[27] Resnicow K, Catley D, Goggin K, Hawley S, Williams GC (2022). Shared decision making in health care: theoretical perspectives for why it works and for whom. Med Decis Making.

[28] Deci EL, Ryan RM (1985). Intrinsic Motivation and Self-Determination in Human Behavior.

[29] Resnicow K, Teixeira PJ, Williams GC (2017). Efficient allocation of public health and behavior change resources: the “difficulty by motivation” matrix. Am J Public Health.

[30] Fagot E (2020). Closing the gap: actualising shared decision-making through effective medication abortion patient follow-up care. BMJ Open Qual.

[31] Frederico M, Michielsen K, Arnaldo C, Decat P (2018). Factors influencing abortion decision-making processes among young women. Int J Environ Res Public Health.

[32] Hooper MW (2024). From distrust to confidence: can science and health care gain what’s missing?. Pew Research.

[33] Noy C (2008). Sampling knowledge: the hermeneutics of snowball sampling in qualitative research. Int J Soc Res Methodol.

[34] Fusch PI, Ness LR (2015). Are we there yet? Data saturation in qualitative research. Qual Rep.

[35] Sandelowski M, Given LM (2008). The SAGE Encyclopedia of Qualitative Research Methods.

[36] Braun V, Clarke V (2023). Toward good practice in thematic analysis: avoiding common problems and be(com)ing a knowing researcher. Int J Transgend Health.

[37] Bans on specific abortion methods used after the first trimester. Guttmacher Institute.

[38] Partial-birth abortion. Legal Information Institute.

[39] Smith KL, Shipchandler F, Kudumu M, Davies-Balch S, Leonard SA (2022). “Ignored and invisible”: perspectives from Black women, clinicians, and community-based organizations for reducing preterm birth. Matern Child Health J.

[40] Mitchell E (2022). Black women’s reproductive health and legacies of distrust. Black Perspectives.

[41] Nguyen M, Cartwright AF, Upadhyay UD (2023). Fear of procedure and pain in individuals considering abortion: a qualitative study. Patient Educ Couns.

[42] Steinberg JR, Tschann JM, Furgerson D, Harper CC (2016). Psychosocial factors and pre-abortion psychological health: the significance of stigma. Soc Sci Med.

[43] Brown SL, Salmon P (2019). Reconciling the theory and reality of shared decision-making: a “matching” approach to practitioner leadership. Health Expectations.

[44] Charles C, Gafni A, Whelan T (1997). Shared decision-making in the medical encounter: what does it mean? (Or it takes at least two to tango). Soc Sci Med.

[45] Chandra S, Mohammadnezhad M, Ward P (2018). Trust and communication in a doctor- patient relationship: a literature review. J Healthcare Commun.

[46] Winny A (2024). How abortion trigger laws impact mental health. Johns Hopkins Bloomberg School of Public Health.

[47] Krafft PM, Spiro ES Keeping rumors in proportion: managing uncertainty in rumor systems.

