# Supplementary material for: Investigating the Impact of Online Abortion Myths on Health Care Providers and Advocates: Interview Study
Source: JMIR Public Health Surveill. 2026 Sep 16;12:e77050. doi: 10.2196/77050 (PMC13580605; doi:10.2196/77050)
Supplement: Supplementary file 1 [file publichealth-v12-e77050-s001.docx]

**Introduction:** Thank you again for your time today. The purpose of this study is to gain your insights into the impacts of false and misleading information related to abortion. At this early stage of this work, we hope to establish a baseline understanding of how particular myths about abortion and related reproductive healthcare emerge for providers and advocates. As misinformation researchers, we are aware of the prevalence of health misinformation, but aim to better understand the broader impacts on clinical practice to work toward interventions to combat misleading information and ensure practitioners can meet patient needs.

**Recording, Transcription, and Anonymization**: All interviews, unless otherwise arranged, will be conducted via Zoom. At our agreed upon interview date/time, we ask that you consent to be recorded. This recording will be transcribed and anonymized by a member of our research team, and we will remove all potentially identifying information from the transcript, unless you request otherwise. Transcripts will be analyzed by researchers affiliated with the UW Center for an Informed Public (CIP) and data will be stored securely on CIP servers. Fully anonymized transcripts may be analyzed by CIP affiliated researchers to generate findings for this and future related projects. Findings will be used to directly inform our needs assessment as proposed in our Population Health Initiative Tier 1 pilot grant, which will lead to additional stages of our project including grant applications and research manuscripts. Findings may be used for additional outputs such as—but not limited to—publications, conference presentations, and future research partnerships. You can withdraw your participants, and all associated data, at any point.

**Consent:** You will be asked to provide verbal consent for your participation at the time of the interview. If you have any additional questions or concerns, please contact a member of our research team.
