# Supplementary material for: Investigating the Impact of Online Abortion Myths on Health Care Providers and Advocates: Interview Study
Source: JMIR Public Health Surveill. 2026 Sep 16;12:e77050. doi: 10.2196/77050 (PMC13580605; doi:10.2196/77050)
Supplement: Supplementary file 2 [file publichealth-v12-e77050-s002.docx]

**Introduction:** Thank you again for your time today. The purpose of this study is to gain your insights into the impacts of false and misleading information related to abortion. At this early stage of this work, we hope to establish a baseline understanding of how particular myths about abortion and related reproductive healthcare emerge for providers and advocates. As misinformation researchers, we are aware of the prevalence of health misinformation, but aim to better understand the broader impacts on clinical practice to work toward interventions to combat misleading information and ensure practitioners can meet patient needs.

**Expectations:** There are many questions we would like to ask and we will get through as many as possible in the time we have together. You are free to skip any question or pause the interview at any time. As a reminder, I will be audio recording our conversation today. If you want to stop the recording at any time, please let me know. Any information that you share today will be kept strictly confidential, and your name and any identifiable information will be stored securely and removed from any public-facing outputs, unless you request otherwise. May I begin recording now?

**Basic Questions**

1. Could you please state your name, job title, and where you work?
a. [If clinician...] If you feel comfortable sharing, where did you receive your medical training
2. [If Advocate] How does the work that you do intersect with abortion care? / [If Medical Provider] Do you see patients with unplanned pregnancy?
a. What counselling options do you provide patients with unplanned pregnancy? --
b. What information about abortion do you include in counselling options? --
c. Does your work involve direct interaction with patients?
i. [Potential follow-up] What does that interaction look like?
ii. [Potential follow-up] [If they do...] How do you seek out the most up-to-date information or training related to abortion care?
d. Is educating people about abortion part of the work that you do?
e. How does the state(s) you work in shape your work within abortion care?
i. [Potential follow-up] If you have worked in multiple states, how have your experiences differed?

**Abortion Myths: Scope**

1. What do you see as the major gaps in public knowledge about abortion?
2. What are some of the most common misconceptions about abortion that you’ve heard in your interactions with patients and/or conversations with peers?
a. When or how do these come up?
b. Have these misconceptions changed after the overturning of *Roe v. Wade,* if so, how?
c. Do patients ever share why these misconceptions resonate with them?
d. Do you have any sense for why these resonate?
3. What are the most common questions patients ask you about related to abortion?

**Information Seeking / Communication**

1. Where do you typically get the most up-to-date information and training about abortion and related care—both medical information and legal, social, etc.?
2. Where have you seen false or misleading information being spread / Where do you think patients are getting their information?
3. What role do you think social media plays in spreading [good / bad] information about abortion?
a. [Potential follow-up] What role do you think social media should play in providing accurate information or countering misleading information about abortion?
4. What information sources do you or would you recommend to a patient?

**Abortion Myths: Impacts**

1. What have you done when a patient has come to you with a misunderstanding about abortion—for example, you mentioned [common misconception mentioned earlier]?
a. If you correct misconceptions like this, what strategies have you used or found most effective?
b. Are there any particular resources you find useful to share?
c. [If they say no/ haven’t corrected, ask] Can you tell us more about how that conversation went? Is there any resource/strategy you would have found useful in that interaction?
2. How has false or misleading information about abortion impacted your work?
a. [Potential follow-up] Has any of this impacted you personally or emotionally?
3. Can you reflect on the effects of false or misleading information about abortion? How does this impact medical training?
4. From your perspective, where is the impact of misinformation most critical? Do you have any suggestions for future research into abortion myths that you would like to see taken up?
